# Delirium Induced by Quetiapine and the Potential Role of Norquetiapine

**DOI:** 10.3389/fnins.2019.00886

**Published:** 2019-08-20

**Authors:** Filipe Almeida, Elisabete Albuquerque, Ilda Murta

**Affiliations:** ^1^Department of Psychiatry, The Coimbra Hospital and University Centre, Coimbra, Portugal; ^2^Faculty of Medicine, University of Coimbra, Coimbra, Portugal

**Keywords:** delirium, quetiapine, anticholinergic, antipsychotics, adverse effects

## Abstract

Quetiapine in an atypical antipsychotic drug that is frequently used for delirium and behavioral and psychological symptoms in dementia. However, its potential anticholinergic effects, mediated primarily through its metabolite norquetiapine, could present as counterproductive adverse effects in these situations. There is little data published discussing this potential negative impact on quetiapine’s safety and tolerability, especially in the elderly. Here, we present what is, to our knowledge, the first published case report of delirium apparently induced by low-dose quetiapine, in a 95-year-old patient with no prior history of mental illness, and the potential role of its metabolite norquetiapine.

## Introduction

Delirium is an acute disorder of attention and cognition, more frequent in elderly people (i.e., those aged 65 years or older) that is common, serious, costly, under-recognized, and often fatal. Cognitive assessment and history of acute onset of symptoms are necessary for diagnosis. Delirium offers opportunities to elucidate brain pathophysiology – it serves both as a marker of brain vulnerability with decreased reserve and as a potential mechanism for permanent cognitive damage (Inouye et al., 2014). The pathophysiology of delirium remains poorly understood as it involves multifactorial dynamic interactions between a diversity of risk factors, each one increasing the risk of delirium only marginally (Cerejeira et al., 2010). The exact mechanism of how direct brain insults and aberrant stress responses lead to the brain dysfunction is not yet fully understood (Maclullich et al., 2013). Nevertheless, many neurotransmitters are potentially implicated in the pathophysiology of delirium, but cholinergic dysfunction is one of the most frequently linked to delirium pathophysiology (Hshieh et al., 2008), correlates with the adverse effects of anticholinergic drugs (Lauretani et al., 2010), and has been proposed as a “final pathway” to delirium regardless of the initial insult (Trzepacs, 2000). Other hypothesis concerning delirium pathophysiology include oxidative metabolism, dysfunction of other neurotransmitters, abnormal signal transduction, changes in blood-brain barrier permeability, endocrine abnormalities and increased inflammatory response (Maldonado, 2008).

Quetiapine is a widely used atypical antipsychotic, a dibenzothiazepine derivative, available in two different pharmaceutical forms: immediate-release (IR) and extended-release (XR). It is extensively metabolized by the liver into various metabolites and only 1% is excreted in the unmetabolized form, in urine. N-desalkylquetiapine, also known as norquetiapine, is the major active metabolite of quetiapine and is produced by the action of isoenzyme CYP34A in cytochrome P450 (López-Muñoz and Álamo, 2013). As of April 2019, its use is approved by the Food and Drugs Administration and several European regulatory agencies for schizophrenia, bipolar disorder (manic and depressive episodes, as well as maintenance treatment) and as add-on treatment in major depressive disorder (MDD). It is used off-label for conditions such as: generalized anxiety disorder, as monotherapy for MDD, as add-on treatment for obsessive-compulsive disorder, for psychosis in Parkinson’s disease, for psychosis and agitation in dementia, and in low-dose for insomnia (Maglione et al., 2011). It has also been proposed for managing the symptoms of delirium (Hawkins et al., 2013).

As anticholinergic drugs have been implicated in the etiology and pathophysiology of delirium as well as in serious adverse effects in dementia, Anticholinergic Risk Scales (ARS) have been developed from pharmacological and epidemiological studies to guide clinical practice, especially in elderly people. In this field, available studies disagree as to whether quetiapine poses low (Rudolph et al., 2008), (Ehrt et al., 2010), moderate (Han et al., 2008), or high (Boustani et al., 2008) anticholinergic risk.

## Materials and Methods

We describe the case of a 95-year-old woman, with no prior cognitive complaints reported by her relatives, who developed delirium after starting quetiapine IR 100 mg at night, for treatment of chronic insomnia. A thorough literature search was performed using PubMed, Medline, Ovid, Embase, and Google Scholar about the existence of other possible cases of delirium induced by quetiapine in the circumstances we describe, without finding similar cases. A review of cases in different circumstances of delirium associated with quetiapine was made, as well as review of the pharmacological properties of quetiapine and norquetiapine.

A written informed consent was obtained from the patient for the publication of this case report.

## Case Presentation

Mrs. I, a 95-year-old woman with no prior history of mental illness, presented to the emergency room (ER) in the afternoon of the 12th March 2018 with a syndrome characterized by confusion and marked psychomotor agitation, with a fluctuating course and sudden onset 2 days before. She had previous, non-recent, diagnoses of hypertension, trigeminal neuralgia and anemia, and for that she was regularly on enalapril 5 mg/day, carbamazepine 200 mg/day, iron sulfate 329.7 mg/day and folate 5 mg/day. She had had a hip fracture in 2017 that was already fully consolidated and had been treated in January 2018 for heart failure – in these episodes, she seems to have developed some symptoms of delirium (fluctuating disorientation, for example), but there was no need for specialized psychiatric observation. She was also recently medicated (1 months before) with diazepam 10 mg at night and (3 days before) with quetiapine IR 100 mg at night, prescribed by her general practitioner due to complaints of chronic insomnia, reported by relatives after they started taking care of the patient after her hip fracture.

Physical and neurological examinations were unremarkable and there where no signs or symptoms of pain or respiratory distress. A complete blood count, urea and electrolytes, liver function tests, glucose, and C-reactive protein (CRP) were also unremarkable. An electrocardiogram and a urine test strip were also unremarkable. After that, she was observed by a psychiatrist, who recommended risperidone 0.5 mg/day and that she kept taking diazepam and quetiapine as before.

That night, and after administering risperidone 0.5 mg, quetiapine 100 mg, and diazepam 10 mg, her relatives brought her back to the ER for increased agitation and maintained confusion, with temporal disorientation. The physical examination was again unremarkable. Complete blood count, urea and electrolytes, liver functions tests, glucose and CRP were again asked for and unremarkable. Another urine test strip was also negative for nitrites, leucocytes or blood. As there was no signs of infection or pain, she was again observed by a psychiatrist. The psychiatrist noted temporal and spatial disorientation, incoherent speech and apparent visual hallucinations. A computed tomography (CT scan) of the head was then performed, which showed no signs of recent intracranial lesions. Of note, there were only bilateral inferior lenticular hypodensities, suggestive of enlarged perivascular spaces, but no signs of leucoencephalopathy or cortical atrophy. Meanwhile, in spite of going against standard clinical practice, a total of 20 mg of haloperidol (intramuscular) were administered throughout 2 days while under observation by several ER teams, due to severe behavioral disturbance, visual hallucinations and refusal to take medication.

In the absence of any neurological or systemic illness which could explain the sudden onset of the symptoms described, the psychiatrist reviewed the patient’s history with the patient’s relatives. A chronological sequence was made clear, with onset of symptoms the day after the patient started quetiapine 100 mg at night. A formal diagnosis of delirium induced by quetiapine was made (meeting criteria established in the *Diagnostic and Statistical Manual for Mental Disorders, Fifth Edition*) and due to the severity of behavioral disturbance, the patient was committed to the Psychiatry ward. Here, quetiapine, risperidone and diazepam were stopped, and she was medicated by the psychiatrist in charge of her case with haloperidol 3 mg/day and clonazepam 0.5 mg/day. After 6 days, she was discharged with no remarkable signs or symptoms at mental state examination and no apparent cognitive deficits.

## Discussion

### Previous Case Reports and Case Series of Delirium Associated With Quetiapine

Reports of quetiapine-induced delirium are rare, contrasting with the extensive data available on other antipsychotics and antidepressants with anticholinergic profiles. Here follows a review of the available case reports and case series on this issue.

Sim et al. (2000), reported the case of a 62-year-old man with schizophrenia who, 3 days after starting quetiapine at a dose of 600 mg/day, developed signs and symptoms of inattention, visual illusions and sleep-wake reversal. An EEG was performed and showed generalized slowing suggestive of metabolic encephalopathy. The symptoms resolved 48 h after stopping quetiapine.

In Balit et al. (2003) published a case series of 45 patients who had overdosed on quetiapine. 3 developed delirium after taking 4–12 g of quetiapine alone and, of these, 2 had to be placed in an intensive care unit (ICU). There was another case report of delirium induced by quetiapine overdose by Alexander (2009), describing the case of a 23-year-old woman who had taken 12 g of quetiapine. Finally, regarding reports of delirium linked to quetiapine overdose, Rhyee et al. (2010) reported the case of a 15-year-old adolescent girl who had overdosed on unknown amounts of quetiapine, clonidine, trazodone and risperidone and responded partially to administration of physostigmine after a tentative diagnosis of anticholinergic delirium attributable to quetiapine was made.

More recently, Miodownik et al. (2008) a case of delirium induced by combined treatment with lithium and quetiapine of a patient with schizoaffective disorder, but the hypothesis presented by the authors to explain this case is of a pharmacodynamic interaction between quetiapine and lithium that increases the brain concentration of the former. Later, in Huang and Wei (2010) present the hypothesis of anticholinergic delirium to explain two cases of delirium in patients with bipolar disorder who had started combinations of quetiapine (a single dose of 100 mg in the first case and 300 mg/day in the second) and sodium valproate (1000 mg/day in both cases). The patients were 53 and 63-year-old and both had mild renal insufficiency secondary to long-term lithium treatment. In this report, the proposed mechanism for delirium was a direct anticholinergic effect of quetiapine or a synergistic effect of quetiapine and sodium valproate in the presence of mild renal insufficiency, that could have increased serum concentrations of quetiapine.

From our review, there are no other reports of delirium linked to quetiapine. However, we found reports of clear anticholinergic side effects in patients taking quetiapine, such as urinary retention (Dharmarajan et al., 2017).

### Norquetiapine as a Putative Mediator of Anticholinergic Side Effects

Receptor binding affinities studies (Bymaster et al., 2003; Shapiro et al., 2003; Jensen et al., 2008), shown on Table 1, are helpful when comparing the muscarinic antagonism potencies of several frequently used antipsychotics – although there are other important variables, such as ligand residence time. From these, unmetabolized quetiapine seems to have limited anticholinergic properties. Nevertheless, quetiapine is extensively metabolized in the liver and 20 metabolites have been identified. The major active metabolite is N-Desalkylquetiapine, or norquetiapine, which has been implicated in the clinical effects of quetiapine. Jensen et al. (2008), for example, proposed that its unique properties as a potent norepinephrine reuptake transporter and partial 5-HT_1__*A*_ might mediate quetiapine’s efficacy in depression, but also described medium- to high-affinity to muscarinic receptors as an antagonist, contrasting with quetiapine is this regard.

**TABLE 1:** Binding affinity of atypical and typical antipsychotic drugs for human muscarinic receptors in clonal cells (Ki – ligand binding affinity, expressed as maximal receptor occupancy according to ligand concentration, in nM).

**Antipsychotics**	**M1 (Ki)**	**M2 (Ki)**	**M3 (Ki)**	**M4 (Ki)**	**M5 (Ki)**
Quetiapine (Bymaster et al., 2003)	120 ± 35	630 ± 230	1320 ± 80	660 ± 100	2990
Norquetiapine (Jensen et al., 2008)	39	453	23	110	23
Aripiprazole (Shapiro et al., 2003)	6780	3510	4680	1520	2330
Chlorpromazine (Bymaster et al., 2003)	25 ± 3	150 ± 14	67 ± 4	40 ± 3	42 ± 2
Haloperidol (Bymaster et al., 2003)	>10000	>10000	>10000	>10000	>10000
Olanzapine (Bymaster et al., 2003)	2.5 ± 0.3	48	13 ± 0.8	10 ± 0.6	6 ± 0.8
Risperidone (Bymaster et al., 2003)	>10000	>10000	>10000	>10000	>10000

Norquetiapine is produced by the CYP3A4 isoform metabolization of quetiapine but is, is turn, primarily metabolized by the CYP2D6 isoform (Bakken et al., 2012). It is interesting to note that carbamazepine, which was part of the patient’s regular medication described in this case report, is a strong inducer of CYP3A4 but not of CYP2D6 (Lynch and Price, 2007) and that its specific action promoting the metabolization of quetiapine to norquetiapine has been described (Grimm et al., 2006).

As we have seen, anticholinergic risk studies have been inconsistent as to whether quetiapine present a low-, moderate- or high-risk, in spite of having been linked to greater cognitive decline in Alzheimer’s disease by Ballard et al. (2005) The pharmacokinetic variability of quetiapine in patients has been established and also correlated with age and CYP3A4 inducers (Bakken et al., 2011), as well as tentatively linked to therapeutic effects (Rovera et al., 2017). We could not find studies associating this variability with the incidence of side effects as there are regarding therapeutic effects.

Our hypothesis for the case described in this paper is that delirium was made much more likely by a pre-existing vulnerability – due to the patient’s advanced age and comorbidities, recent episodes with symptoms of delirium and prescription of diazepam – but that a tipping point was reached after the introduction of quetiapine, whose metabolization to norquetiapine, a more potent muscarinic antagonist, is promoted by carbamazepine, with which the patient was also medicated.

## Conclusion

With this report, we aim to draw attention to a cluster of potential side effects of quetiapine, which have been overlooked by available studies. We suggest that variability in quetiapine’s conversion to norquetiapine could be one of the factors involved in the inconsistency of reports regarding quetiapine’s side effects due to its anticholinergic action. Individual patient characteristics and drug interactions contribute to this variability and should be accounted for when assessing anticholinergic risk profiles.

## Data Availability

All datasets generated for this study are included in the manuscript and/or the supplementary files.

## Author Contributions

FA performed the review, wrote the manuscript, and incorporated feedback from all co-authors. EA and IM contributed substantially to drafting the article or revising it critically for important intellectual content. All authors had given the final approval of the version to be published.

## Conflict of Interest Statement

The authors declare that the research was conducted in the absence of any commercial or financial relationships that could be construed as a potential conflict of interest.
